# SOCIAL ISOLATION AND WELL-BEING IN OLDER ADULTS: EXAMINING THE POTENTIAL PROTECTIVE EFFECT OF PHYSICAL ACTIVITY

**DOI:** 10.1093/geroni/igae098.2544

**Published:** 2024-12-31

**Authors:** Babatope Ogunjesa, Sadia Anjum Ashrafi, Rifat Alam, Emmanuel Dubure, Andiara Schwingel

**Affiliations:** University of Illinois Urbana-Champaign, Champaign, Illinois, United States; University of Illinois Urbana-Champaign, Champaign, Illinois, United States; University of Illinois Urbana-Champaign, Champaign, Illinois, United States; University of Illinois Urbana-Champaign, Champaign, Illinois, United States; University of Illinois Urbana-Champaign, Champaign, Illinois, United States

## Abstract

As individuals age, the issue of loneliness and social isolation becomes a growing concern for public health. These can have significant health consequences for older adults, including an elevated risk of heart disease, high blood pressure, depression, and dementia. To explore the association between social isolation and self-reported general health as well as the interaction of physical activity, we conducted a study analyzing a sample of 2,203 older adults (aged 65 and above) from the Health Information National Trends Survey 6 (HINTS 6). We used a logistic regression model while controlling for socio-demographic factors. Our study found that older adults with high levels of social isolation are less likely to report high self-reported general health (OR=0.958, 95%CI=0.931 – 0.987). However, those who met the recommended guideline of 150 minutes or more per week of moderate-vigorous physical activity are more likely to experience better self-reported general health (OR=3.37, 95%CI=1.878 – 6.050) than those who don’t meet the guideline. In our interaction effect analysis, we observed that for both physical activity groups, the predicted general health outcome decreases over time with an increased isolation score, but it decreases faster for those getting less than 150 minutes of moderate exercise per week. These findings suggest that social isolation has negative impact on health in later life and that regular moderate physical activity and meeting recommended guidelines may help protect older adults against the adverse health consequences of isolation. Interventions targeting social connectivity and promoting physical activity may improve isolated older adults’ health outcomes.

